# Clostridioides difficile Infection in Inflammatory Bowel Disease Patients: A Systematic Review of Risk Factors and Approach in Management

**DOI:** 10.7759/cureus.43134

**Published:** 2023-08-08

**Authors:** Leslie Sangurima, Maujid Masood Malik, Nency Ganatra, Rosemary Siby, Sanjay Kumar, Sara Khan, Doju Cheriachan, Lubna Mohammed

**Affiliations:** 1 Internal Medicine, California Institute of Behavioral Neurosciences and Psychology, Fairfield, USA; 2 Biomedical Sciences, King Faisal University, Hofuf, SAU; 3 Internal Medicine, Bahria University Medical and Dental College, Pakistan Navy Ship (PNS) Shifa Hospital, Karachi, PAK; 4 Emergency Medicine, California Institute of Behavioral Neurosciences and Psychology, Fairfield, USA

**Keywords:** risk factors, ulcerative colıtıs, chron’s disease, clostridioides difficile infection, inflammatory bowel disease

## Abstract

*Clostridioides difficile* infection (CDI) is one of the most common diseases associated with medical care, having a more significant impact on patients with inflammatory bowel disease (IBD). The latest studies have proposed a change in management for CDI in IBD patients. This study aims to perform a systematic review that explores the risk factors associated with the infection and the most optimal approach in management. Multiple databases were used for this research, including PubMed, Google Scholar, Science Direct, and Cochrane Library. Studies published in the last five years in the English language were selected based on pre-established criteria. The quality assessment used was the Assessment of Multiple Systematic Review, the Newcastle-Ottawa Scale, and the Scale for the Assessment of Narrative Review Articles.

Twelve studies met the inclusion criteria in this systematic review, including literature reviews, a case and control study, and systematic reviews and meta-analyses. Based on the findings in this research, we conclude that the treatment for an initial episode of CDI in IBD patients is the use of antibiotics, vancomycin, or fidaxomicin. For episodes of recurrent CDI (rCDI), fetal microbiota transplantation should be considered. The most common risk factors associated are gut microbiota disturbances, the use of antibiotics, and hospitalization. Due to a wide range of risk factors mentioned in some studies but disregarded in others, further research is needed to determine the most prevalent risk factors.

## Introduction and background

*Clostridioides difficile* (*C. difficile*) is one of the most common microorganisms associated with medical care infection in the United States [1]. It is a major health problem, with an estimated 223,900 cases reported in hospitalized patients and 12,800 deaths around the United States in the year 2017 [2]. Risk factors for infections include age above 65 years old, prolonged hospital stays, recent use of broad-spectrum antibiotic therapy, and the presence of comorbidities like inflammatory bowel disease (IBD) [3]. Infection-associated diseases are caused by colonization of the bacteria followed by toxin production, which varies its clinical manifestation depending on the type of strain and the immune status of the host. The strains can be differentiated by REA typing, polymerase chain reaction, and toxinotyping studies. The two main pathogenic strains are endotoxins classified as toxins A (tcdA) and B (tcdB) [4]. Recent studies report that *C. difficile* infections (CDI) are turning more severe and resistant to antibiotics, resulting in a mortality rate of 1-2% in these cases [3]. Antibiotic treatment for CDI is usually followed by recurrent infection from the same pathogen, resulting in the use of alternative treatments such as fecal microbiota transplantation (FMT) and oral ingestion of nontoxigenic *C. difficile* spores [1].

IBD is an immune-mediated chronic relapsing disease consisting of two subtypes: Crohn’s disease (CD) and ulcerative colitis (UC). It is thought to be the result of interactions between the environment, microbiome, and immune-mediated factors in a susceptible host [5]. Treatment for this disease involves corticosteroids, immunomodulators, biologic therapy, and antibiotics with the knowledge that some of the therapies can increase the risk of acquiring CDI [6]. The majority of IBD patients seem to have acquired CDI in a community setting, as shown in a study with 47.2% of patients infected this way [6]. This infection can occur with gastrointestinal symptoms indistinguishable from an exacerbation of IBD; for this reason, routine screening has been recommended for an acute flare associated with diarrhea in an IBD patient [7]. The American College of Gastroenterology (ACG) has implemented in their guidelines that immunosuppressive therapy for IBD should not be held during CDI infection in the backdrop of disease flare, and an escalation of therapy may be an option if there is no symptomatic improvement with CDI treatment [5].

Even with limited data comparing CDI in IBD and non-IBD patients, there is a correlation with IBD patients having a more aggressive clinical course with up to six times more probability of undergoing bowel surgery compared to non-IBD patients [8]. To address the gaps and to update our understanding of risk factors and management associated with CDI in patients with IBD, we have elaborated on this systematic review with the goal of using this information to improve prevention by acknowledging common risk factors and treatment intervention.

## Review

Methods

The Preferred Reporting Items for Systematic Review and Meta-Analysis (PRISMA) guidelines 2020 were used to perform this systematic review, and the population, intervention, and outcome (PIO) framework was included in this study [9,10].

Eligibility Criteria

Inclusion criteria for this study include relationships with the topic with adult human subjects only and original publication in the English language within the last five years (2017-2022), where the free full-text article is available or can be obtained through the authors. The studies selected include randomized controlled trials, systematic reviews, observational studies, and meta-analyses that included (1) the study population of adult IBD patients with CDI of all races; (2) the use of antibiotic, FMT, and biologic therapy; and (3) primary outcomes in the initial and general cure rates with selected treatments. Non-English language articles published before 2017, animal studies, book articles, and grey literature were excluded from this study.

Database and Search Strategy

The Pubmed, Google Scholar, Science Direct, and Cochrane Library databases were used to perform a comprehensive search from 2017 to 2022. We used appropriate keywords and Medical Subject Heading (MeSH) terms to obtain relevant articles describing the association between IBD and *C. difficile*. The descriptions used for each database can be seen in Table 1.

**Table 1:** A description of search strategies used in PubMed, Google Scholar, Science Direct, and Cochrane Library *C. difficile*: *Clostridioides difficile*, IBD: inflammatory bowel diseases

Database	Keywords	Search strategy	Filters	Search result
PubMed	*C. difficile* OR *Clostridioides difficile* OR *Clostridium difficile* AND inflammatory bowel disease	*C. difficile* OR *Clostridioides difficile* OR *Clostridium difficile* OR ("*Clostridioides difficile*/analysis"[Mesh] OR "*Clostridioides difficile*/classification"[Mesh] OR "*Clostridioides difficile*/drug effects"[Mesh] OR "*Clostridioides difficile*/etiology"[Mesh] OR "*Clostridioides difficile*/growth and development"[Mesh] OR "*Clostridioides difficile*/immunology"[Mesh] OR "*Clostridioides difficile*/microbiology"[Mesh] OR "*Clostridioides difficile*/pathogenicity"[Mesh] OR "*Clostridioides difficile*/physiology"[Mesh] ) AND inflammatory bowel disease OR IBD OR ( "inflammatory bowel diseases/analysis"[Mesh] OR "inflammatory bowel diseases/anatomy and histology"[Mesh] OR "inflammatory bowel diseases/complications"[Mesh] OR "inflammatory bowel diseases/diagnosis"[Mesh] OR "inflammatory bowel diseases/diet therapy"[Mesh] OR "inflammatory bowel diseases/drug therapy"[Mesh] OR "inflammatory bowel diseases/epidemiology"[Mesh] OR "inflammatory bowel diseases/etiology"[Mesh] OR "inflammatory bowel diseases/immunology"[Mesh] OR "inflammatory bowel diseases/microbiology"[Mesh] OR "inflammatory bowel diseases/physiopathology"[Mesh] OR "inflammatory bowel diseases/prevention and control"[Mesh] OR "inflammatory bowel diseases/therapy"[Mesh] OR "inflammatory bowel diseases/transmission"[Mesh])	Free full text, five years, humans, English, adult 19+ years, meta-analysis, randomized controlled trial, systematic review	346
Google Scholar	Inflammatory bowel disease and *Clostridioides difficile* and *Clostridium difficile*	Inflammatory bowel disease and *Clostridioides difficile* and *Clostridium difficile*	2017-2022	90
Science Direct	Inflammatory bowel disease and *Clostridium difficile* and *Clostridioides difficile*	Inflammatory bowel disease and *Clostridium difficile* and *Clostridioides difficile*	2017-2022, review article, research articles, case reports, open access only	115
Cochrane Library	Inflammatory bowel disease	Inflammatory bowel disease	2017-2022, infectious disease	2

All references that were obtained through the databases were imported to Microsoft Excel 2021 (Microsoft, Redmon, Washington) to eliminate duplicates.

Risk of Bias

We assessed the remaining full articles for the potential risk of bias using the following tools with their respective study: Assessment of Multiple Systematic Review 2 (AMSTAR 2), Newcastle-Ottawa Scale (NOS), and Scale for the Assessment of Narrative Review Articles 2 (SANRA 2). Two authors (Leslie Sangurima and Maujid Masood Malik) independently reviewed the quality of the studies, with each evaluation instrument requiring a minimum score of 70% to be accepted. Table 2 indicates the quality appraisal tool and its items used.

**Table 2:** Quality assessment of each type of study AMSTAR 2: Assessment of Multiple Systematic Reviews 2, NOS: Newcastle-Ottawa Scale, SANRA 2: Scale for the Assessment of Narrative Review Articles

Quality assessment tool	Type of study	Items and their characteristics	Total score	Accepted score (70%)	Accepted studies
AMSTAR 2 (5)	Systematic reviews and meta-analyses	Sixteen items: 1. Did the research questions and inclusion criteria for the review include components of PICO? 2. Did the report of the review contain an explicit statement that the review methods were established prior to the conduct of the review and did the report justify any significant deviations from the protocol? 3. Did the review authors explain their selection of the study designs for inclusion in the review? 4. Did the review authors use a comprehensive literature search strategy? 5. Did the review authors perform study selection in duplicate? 6. Did the review authors perform data extraction in duplicate? 7. Did the review authors provide a list of excluded studies and justify the exclusions? 8. Did the review authors describe the included studies in adequate detail? 9. Did the review authors use a satisfactory technique for assessing the risk of bias (ROB) in individual studies that were included in the review? 10. Did the review authors report on the sources of funding for the studies included in the review? 11. If meta-analysis were performed did the review authors use appropriate methods for the statistical combination of results? 12. If meta-analysis was performed, did the review authors use to assess the potential impact of RoB in individual studies on the results of the meta-analysis or other evidence synthesis? 13. Did the review authors account for RoB in individual studies when interpreting/ discussing the results of the review? 14. Did the review authors provide a satisfactory explanation for, and discussion of, any heterogeneity observed in the results of the review? 15. If they performed quantitative synthesis did the review authors carry out an adequate investigation of publication bias (small study bias) and discuss its likely impact on the results of the review? 16. Did the review authors report any potential sources of conflict of interest, including any funding they received for conducting the review? Scored Yes or No, Partial Yes was considered as a point.	16	12	5
NOS (3)	Case-control and cohort studies	8 items: 1. Representativeness of the exposed cohort. 2. Selection of the non-exposed cohort. 3. Ascertainment of exposure. 4. Demonstration that the outcome of interest was not present at the start of the study. 5. Comparability of cohorts on the basis of the design or analysis. 6. Assessment of outcome. 7. Was follow-up long enough for outcomes to occur. 8. Adequacy of follow-up of cohorts. Scored as 0, 1, and 2 points.	8	6	1
SANRA 2 (13)	Narrative review	Six items: 1. Justification of the article’s importance for the readership. 2. Statement of concrete aims or formulation of questions. 3. Description of the literature search. 4. Referencing. 5. Scientific reasoning. 6. Appropriate presentation of data.	12	9	6

Results

**Figure 1:**
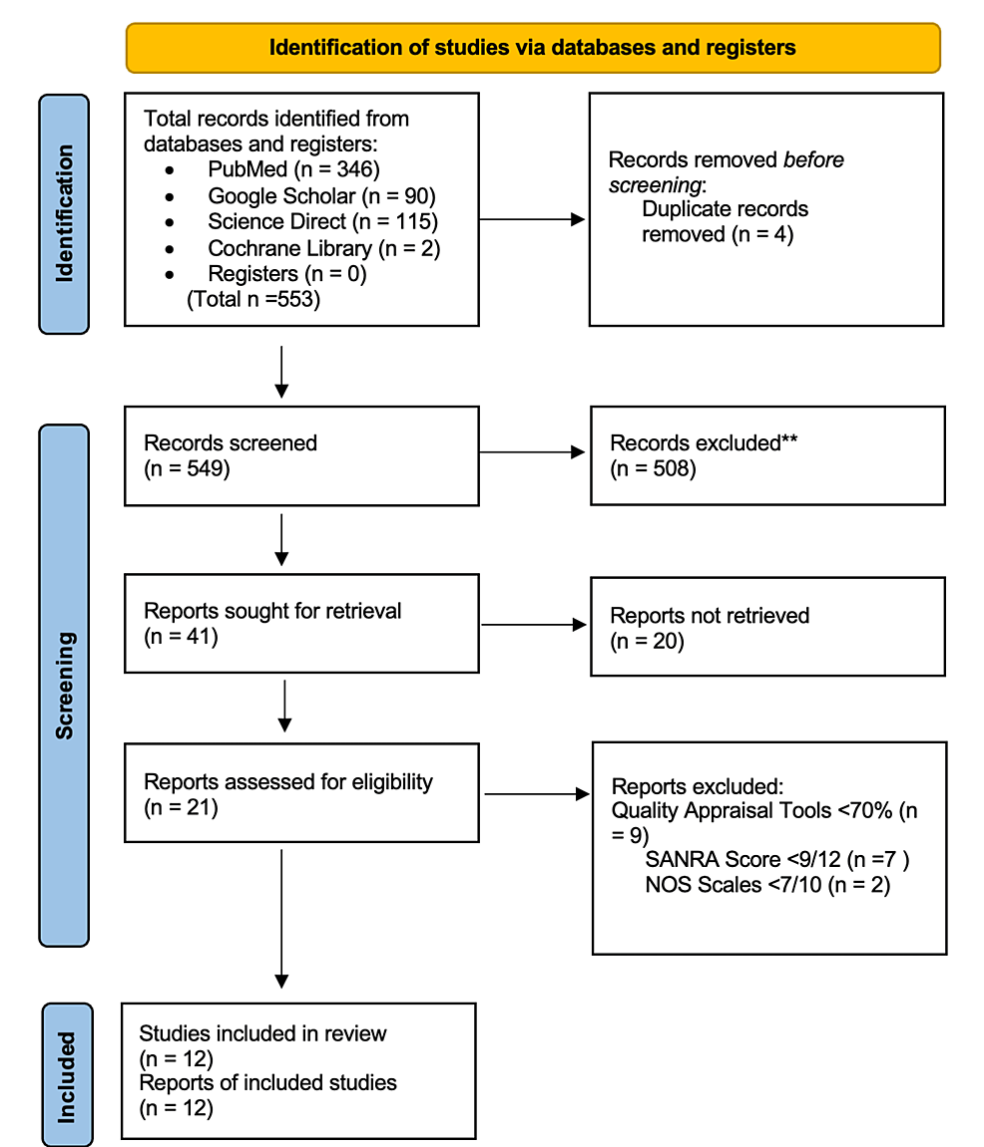
PRISMA flowchart of the study research selection

We have identified a total of 553 records using the previously mentioned search strategies across various databases that were relevant to this systematic review. Out of the 553 records obtained, 346 were accessed through PubMed, 90 from Google Scholar, 115 from Science Direct, and two from Cochrane Library. The records were checked and organized in alphabetical order using Microsoft Excel 2021 (Microsoft, Washington, USA), and then we manually excluded four duplicates. The remaining 553 were thoroughly screened for relevance based on their titles and abstract, during which 508 records were excluded for lacking relevance. An overall of 41 articles were sought for retrieval of which 20 were not obtained due to not being free full text or not receiving articles from authors. After assessing 21 articles for eligibility, nine were removed because of their low quality. A total of 12 articles were used for this systematic review, including systemic reviews/meta-analyses, case-control and cohort studies, and narrative reviews. The complete PRISMA 2020 flow diagram for systematic reviews can be seen in Figure 1.

Study Characteristics

The main characteristics of the 12 studies selected for this systematic review are shown in chronological order in Table 3. There is also a brief mention of the outcome of each study.

**Table 3:** Main characteristics of the studies included in the review CD: Crohn’s disease, CDI: *C. difficile* infection, FMT: fecal microbiota transplantation, IBD: inflammatory bowel disease, NSAID: non-steroidal anti-inflammatory drug, UC: ulcerative colitis

Author	Year	Location	Title	Type of study	Identified risk factors	Management outcomes
Boeriu et al. [11]	2022	Romania	The current knowledge on *Clostridioides difficile* infection in patients with inflammatory bowel disease	Literature review	Increasing age and comorbidities, gut microbiota disturbance, antibiotics use, nonsteroidal anti-inflammatory drugs within two months prior to hospital risk, pancolitis, corticosteroids, and biologic therapies, interleukin-4 single nucleotide polymorphisms	Oral vancomycin 125 mg four times a day for 10 days is the first-line treatment, it also reduces the necessity for colectomy. Due to rates of recurrence after vancomycin, fidaxomicin 200 mg daily for 10 days is used as an alternative for initial treatment. With an increase of hypervirulent strains resistant to traditional management, monoclonal antibodies actoxumab and bezlotoxumab are now used
Del Vecchio et al. [12]	2022	Italy	Risk factors, diagnosis, and management of *Clostridioides difficile* infection in patients with inflammatory bowel disease	Literature review	Antibiotic use, colonic involvement, dysbiotic gut microbiota, NSAID, corticosteroid, and biological therapies	Use of vancomycin and fidaxomicin are the first-line treatment therapies, if they are not available metronidazole is administered. The first recurrence of CDI should be treated with different antibiotic regimens from the first episode. A safer and more tolerable option for recurrent CDI is FMT
Khanna et al. [13]	2021	USA	Management of *Clostridioides difficile* infection in patients with inflammatory bowel disease	Literature review	Antibiotic use, dysbiosis in gut microbiota	Vancomycin and fidaxomicin are recommended as first-line treatment, metronidazole is no longer recommended for the management of CDI. Prevention for recurrence is avoiding risk factors and optimal management of IBD with a goal of remission. Fidaxomicin is used for the first recurrence and to prevent future recurrent CDI FMT is used
Sehgal et al. [14]	2021	USA	The interplay of *Clostridioides difficile* infection and inflammatory bowel disease	Literature review	Dysbiosis in gut microbiota, antibiotic use, immunosuppressive therapy, prior CDI, hospitalization, surgeries, stay in a long-term care facility	In broad terms, there are three treatment options for CDI in IBD patients: pharmacotherapy, FMT, and surgery. The initial episode of CDI is treated with vancomycin or fidaxomicin for 10 days, and metronidazole is not recommended. For the first recurrence, fidaxomicin is used, but its cost is an ongoing challenge. FMT is emerging as the preferred option for recurrent CDI in patients with underlying IBD. The decision of titration of immunosuppression therapy for IBD patients with superimposed CDI is made on an individual basis
Tariq et al. [15]	2021	USA	Outcomes of fecal microbiota transplantation for *C. difficile* infection in inflammatory bowel disease: a systematic review and meta-analysis	Systematic review and meta-analysis	Disruption of healthy gut microbiota	FMT is a safe and effective therapy for CDI, with an overall cure rate higher in multiple FMTs than in a single FMT. Twenty-five percent of patients experienced IBD flare after FMT. Less than 10% of adults underwent colectomy after FMT, most of them due to worsening IBD
Yue et al. [16]	2020	China	Regulation of the intestinal microbiota: an emerging therapeutic strategy for inflammatory bowel disease	Literature review	Dysbiosis in gut microbiota	Treatments targeting intestinal microbiota, probiotics, prebiotics, symbiotics, herbal medicine, and FMT implement therapeutic action by correcting dysbiosis. With current treatment methods offering low effectiveness with the rapid rise in IBD incidence, these complementary and alternative therapies should be considered
Balram et al. [17]	2019	USA	Risk factor associated with *Clostridium difficile* infection in inflammatory bowel disease: a systematic review and meta-analysis	Systematic review and meta-analysis	Antibiotic use, colonic involvement, biologic medication	IBD patients with CDI have an almost fourfold increase in risk of in-hospital and long-term mortality, justifying the need for rapid diagnosis and aggressive treatment. CDI doubled the odds of having colectomy in the long term. It is not associated with a short-term risk of colectomy
Beniwal-Patel et al. [18]	2019	USA	The juncture between *Clostridioides difficile* infection and inflammatory bowel diseases	Literature review	Antibiotic use, restorative procto-colectomy with ileal pouch-anal anastomosis, NSAID within two months prior to admission, history of CD within the past 12 months, emergency department visits 12 weeks prior to admission	For first-time CDI, initial therapy consists of either vancomycin or fidaxomicin. The option to stop immunosuppression therapy has not been well studied, but it is important to consider that biological therapies have very long half-lives, so a cease in therapy would not stop the immunosuppression
Moens et al. [19]	2019	Belgium	*Clostridium difficile* infection in inflammatory bowel disease: epidemiology over two decades	Case and control	Dysbiosis in gut microbiota, colonic involvement, corticosteroids	Vancomycin is recommended as first-line treatment, lower rates of recurrent CDI after vancomycin could not be confirmed in the cohort used. The number of FMT for recurrent CDI was very low due to cases of refractory CDI being excluded as they were not established in the hospital. The association between the risk of colectomy and CDI has yielded inconsistent results with a lot of heterogeneity in the studies observed
Chen et al. [20]	2018	China	Effect of faecal microbiota transplantation for treatment of *Clostridium difficile* infection in patients with inflammatory bowel disease: a systematic review and meta-analysis of cohort studies	Systematic review and meta-analysis	Hospital stays, recurrence despite receiving standard treatment	Usual treatment includes metronidazole or vancomycin results in recurrence rates between 22-34%. The use of FMT normalizes gut dysbiosis, with an initial cure rate of 81% [95% CI= 76-85%], demonstrating it as an effective therapy for CDI in patients with IBD
Chen et al. [21]	2017	Australia	*Clostridium difficile* infection and risk of colectomy in patients with inflammatory bowel disease: a bias-adjusted meta-analysis	Meta-analysis	Dysbiotic gut microbiota, severe IBD flares, elevated surgical rates, colonic involvement, immunomodulatory therapy	Initial management for IBD is medical therapy until there is a failure in treatment or complications occur, which then should be followed by colectomy. UC has higher surgical rates due to it being limited to the colon, compared to CD
D’Aoust et al. [22]	2017	Canada	Management of inflammatory bowel disease with *Clostridium difficile* infection	Systematic review	Broad-spectrum antibiotic exposure, recent hospitalization, immunosuppression, increased age, comorbidities, interleukin-4-associated single nucleotide polymorphism	For Mild to moderate CDI, the use of metronidazole or vancomycin is recommended, when the disease is severe or with complications the use of vancomycin is first-line therapy. For the first recurrence of CDI, treatment includes metronidazole, vancomycin, and fidaxomicin. Subsequent recurrence should consider FMT

Discussion

The relationship between IBD and CDI has always been heavily intertwined, having received more attention these past years with several studies being done on their interaction. From the controversial topic of biological therapy use or when to use metronidazole, the current perception of management is changing. Due to a wide variation of results in determining their risk factors and management approach, we have analyzed recent literature to assist in clarifying the current approach to these topics.

Risk Factors Associated With CDI

Traditional risk factors associated with an increased risk of CDI in IBD patients include increasing age and comorbidities causing a gut microbiota disturbance, reducing its resistance to infection [11]. One of the most known risk factors is the overuse of antibiotics, specifically broad-spectrum antibiotics, due to their inhibiting effect on the growth of normal gut microbiota or directly killing the micro-organisms, which in humans is the main protective mechanism against intestinal infections [12].

The pathogenesis of IBD creates the ideal environment for infection by altering intestinal immunity and its microbiota. Colonic involvement is linked to an increased risk of CDI, affecting 1.5 times more frequently in UC patients compared to CD due to its pathogenesis [12]. In a recent meta-analysis by Balram et al., the medical therapy for IBD also facilitates infection with the use of biologic therapy, doubling the risk of CDI among IBD patients (OR: 1.65, 95% CI: 1.18, 2.30) [17]. In a retrospective study done in Greece by Viazis et al., the prevalence of CDI in patients receiving azathioprine monotherapy was significantly higher than in patients receiving other medication, including corticosteroids and 5-aminosalicylic acid [23].

Other aspects to keep in mind are the genetic and immunologic risk factors; a retrospective cohort study with 172 IBD patients indicates a link with interleukin-4-associated single nucleotide polymorphism (rs2243250) [11,22]. Another research studied the humoral response to toxins A and B produced by the bacteria, finding a weakened ability to generate strong toxin-specific antibodies and B-cell responses could be a factor in developing CDI in IBD patients. Meanwhile, high-serum anti-toxin A or anti-toxin B antibodies are protective against recurrent infections [22]. A retrospective case-control study of 306 IBD patients discovered that cytomegalovirus-infected patients were at a higher risk of being co-infected with the bacteria [22].

The use of NSAIDs has an unclear impact on CDI, with a few studies suggesting it as a risk factor when observed within two months prior to admission and others indicating no effects could be found [18]. In the general population, proton pump inhibitors are associated with an amplified risk of CDI due to the greater rate of transformation of spores into vegetative cells by the reduced acidity in the stomach. This risk does not present with IBD patients because they already present a remarkable dysbiosis in the gut [12].

Antibiotic Management

Management of CDI is shaped by the severity of the disease and the frequency of recurrences. A first-line treatment for non-severe CDI indicated by the ACG recommends the use of oral vancomycin 125 mg four times a day for 10 days or oral fidaxomicin 200 mg twice a day for 10 days [5]. This strategy is also supported by the current clinical guidelines of the Infectious Diseases Society of America and the Society for Healthcare Epidemiology of America published in 2018 [24]. Oral metronidazole 500 mg three times daily for 10 days may be considered, but the therapeutic response is poor [11]. There is also additional data obtained from the use of long duration, between 21 through 42 days of oral vancomycin, which demonstrates a decrease in recurrence rates [12].

For the treatment approach for a first non-fulminant episode of CDI in IBD patients, the ACG suggests oral vancomycin 125 mg four times a day for a minimum of 14 days, otherwise fidaxomicin 200 mg twice daily [5,12]. A retrospective study, elaborated by Horton et al., with 114 non-severe CDI patients registered shorter lengths of stay and fewer readmissions when treated with regimens containing vancomycin in contrast with those treated with metronidazole [25]. One of the most controversial topics with management is the use of immunosuppressive agents, due to their possibility of delaying eradication and its necessity for the underlying IBD [5]. Current guidelines recommend initiating antibiotic therapy while maintaining IBD therapy, and if no clinical improvement is seen after three days, immunosuppressive agents should be escalated for the management of the underlying active IBD [5].

Patients with fulminant CDI, who presents with hypotension, shock, ileus, or megacolon, require an early evaluation and should be treated with adequate volume resuscitation and oral vancomycin 500 mg every six hours daily combined with parenteral metronidazole 500 mg every eight hours daily [12]. This recommendation is based on a retrospective study, where patients with fulminant CDI in the intensive care unit received vancomycin and metronidazole had lower mortality rates compared with only the use of vancomycin (15.9% vs 36.4%, P = 0.03). If ileus is present, the addition of vancomycin enemas may be considered based on multiple guidelines [5].

Recurrent Infection Management

Recurrent CDI (rCDI) is defined as the recurrence of diarrhea and a confirmatory positive test within 8-12 weeks after treatment of an initial episode. Patients with IBD have up to a 30% increased risk for rCDI compared to the general population [12]. Treatment should be tapering or pulsed-dose vancomycin in patients who were initially treated with fidaxomicin, vancomycin, or metronidazole. Fidaxomicin should be recommended in patients experiencing a first recurrence after using vancomycin or metronidazole in the initial course [5].

Prevention strategies for recurrence include avoiding the presence of other risk factors, the use of narrow-spectrum antibiotics for the management of CDI, and maintaining the underlying IBD in remission [13]. The use of bezlotoxumab can be considered as a prevention strategy, and this monoclonal antibody goes against *C. difficile* antitoxin B. Recent clinical trials have demonstrated a 50% relative reduction in recurrence incidence [13].

Management With FMT

The pathophysiology of CDI, especially the recurrent subtype, involves the dysbiosis of gut microbiota with the presence of IBD being considered a risk factor for secondary CDI. A safe and effective management for microbiota restoration is FMT, having an efficacy of over 80-90% prevention of rCDI in non-IBD patients [13]. A retrospective study done by Khoruts et al. compared the efficacy of FMT in patients with IBD and non-IBD. A single colonoscopic FMT resolved CDI in 74.4% of IBD patients and 92.1% in non-IBD patients (P=.0018) [26]. A meta-analysis done by Chen et al. analyzed nine cohort studies with 346 CDI patients with IBD, and the effect of FMT on its treatment determining its initial cure rate was 81% and an overall cure rate of 89%, with no significant difference in cure rates among patients with IBD and non-IBD (P=0.06) [20].

Recent guidelines indicate FMT should be delivered through colonoscopy or capsules for treatment of rCDI, in cases where the procedures are not available enema could be used. Apart from availability, the choice of delivery should also be driven by the preference of the patient and the clinical circumstances [5]. Minor temporary symptoms include bloating, abdominal pain, cramps, nausea, gas, diarrhea, constipation, and low-grade fevers. Thorough donor selection and screening can lower the risk of transmitting infection [5].

Tariq et al. conducted a recent study among 457 adult patients, in which 363 has a resolution of CDI after the first FMT with a pooled cure rate of 78% and with an overall efficacy in adult patients was 88%, similar to results that were reported in non-IBD patients [15]. This study also indicates that adults who fail a single FMT may benefit from multiple FMTs, suggesting that FMT is a highly effective therapy for CDI in IBD patients [15].

Limitations

Our study has several limitations that should be considered. First, after an exhaustive and methodical search of the literature, we only included studies that were published in the last five years with free full-text available articles or obtained through the authors that were in the English language. Secondly, a majority of the studies had patients with different demographic characteristics, hospital settings, clinical features, and CDI management with some of the reports selected having small sample sizes. Thus, results should be interpreted with caution. Nonetheless, this systematic review also has its strengths. We were able to gather more information about the existing relationship between CDI and IBD, and we deem the data shown in this review can influence future studies on this issue.

## Conclusions

This systematic review intended to explore the risk factors of CDI in IBD patients and its current management of treatment of an initial episode of rCDI. CDI in the setting of IBD has been identified as one of the most common complications to occur, leading to a rise in morbidity and mortality rates. Risk factors play an important role in its appearance and future management. These include the use of broad-spectrum antibiotics, dysbiosis in gut microbiota, and associated comorbidities. Initial antibiotic treatment for the first episode of CDI is vancomycin or fidaxomicin, using metronidazole as an alternative if the first two options aren’t available. rCDI should be treated with a tapering dose of vancomycin if the initial episode was treated with vancomycin, fidaxomicin, or metronidazole. Fidaxomicin is used when the initial treatment was vancomycin or metronidazole. FMT should also be considered for the restoration of gut microbiota in cases of rCDI, with recent studies showing great improvements after use. Future prospective studies are required to corroborate these risk factors findings taking into consideration sex and ethnicity and to support guideline management of CDI in IBD patients.
